# Macrophage presence is essential for the regeneration of ascending afferent fibres following a conditioning sciatic nerve lesion in adult rats

**DOI:** 10.1186/1471-2202-12-11

**Published:** 2011-01-20

**Authors:** Ernesto A Aguilar Salegio, Anthony N Pollard, Malcolm Smith, Xin-Fu Zhou

**Affiliations:** 1 Department of Human Physiology and Centre for Neuroscience, Flinders University, GPO Box 2100, Adelaide 5001, Australia

## Abstract

**Background:**

Injury to the peripheral branch of dorsal root ganglia (DRG) neurons prior to injury to the central nervous system (CNS) DRG branch results in the regeneration of the central branch. The exact mechanism mediating this regenerative trigger is not fully understood. It has been proposed that following peripheral injury, the intraganglionic inflammatory response by macrophage cells plays an important role in the pre-conditioning of injured CNS neurons to regenerate. In this study, we investigated whether the presence of macrophage cells is crucial for this type of regeneration to occur. We used a clodronate liposome technique to selectively and temporarily deplete these cells during the conditioning phase of DRG neurons.

**Results:**

Retrograde and anterograde tracing results indicated that in macrophage-depleted animals, the regenerative trigger characteristic of pre-conditioned DRG neurons was abolished as compared to injury matched-control animals. In addition, depletion of macrophage cells led to: (i) a reduction in macrophage infiltration into the CNS compartment even after cellular repopulation, (ii) astrocyte up-regulation at rostral regions and down-regulation in brain derived neurotrophic factor (BDNF) concentration in the serum.

**Conclusion:**

Activation of macrophage cells in response to the peripheral nerve injury is essential for the enhanced regeneration of ascending sensory neurons.

## Background

Injured axons in the mammalian central nervous system (CNS), compared to those in the peripheral nervous system (PNS), do not regenerate. The limited capacity of matured CNS axons to regenerate has been attributed to the presence of an inhibitory barrier formed by myelin and its associated molecules [1], especially when expressed after injury [2,3]. Consequently, many of the damaged axons undergo atrophy and form 'dystrophic end balls' indicative of halted attempts at regeneration [4]. The use of peripheral nerve grafts to circumvent inhibitory cues in the injured CNS environment have been successful at stimulating axonal regeneration [1,5,6] and have provided important evidence for the intrinsic ability of adult injured neurons to regrow and remain in a regenerative mode within the CNS.

In dorsal root ganglion (DRG) neurons, injury to the peripheral branch in the form of a sciatic nerve injury (SNI) prior to injury to the CNS branch (spinal cord - dorsal column cut), has been previously shown to result in axonal regeneration of centrally projecting ascending fibres [7]. This is referred to as conditioning of DRG neurons and it demonstrates the regenerative capacity of injured fibres in the matured CNS. However, the exact mechanism mediating this regenerative trigger is not fully understood. It has been proposed that activation of macrophage cells, together with satellite cell activation and proliferation within the DRG, as part of the normal PNS response to injury, might be playing a crucial role [8].

Interestingly, the exogenous application of cAMP (cyclic adenosine monophosphate) to DRGs *in vivo *can mimic the regeneration of the injured CNS branch, without injury to the peripheral DRG branch [9]. Mechanistically, this regenerative response after cAMP administration, has been attributed to the blocking and/or reduction in sensitivity of axons to myelin inhibitors in the CNS [10]. Elevation in cAMP by priming and/or exposing neurons *in vitro *to trophic factors such as brain derived neurotrophic factor (BDNF) resulted in neurite outgrowth in the presence of inhibitory molecules such as myelin and myelin-associated glycoprotein (MAG) [11]. This is important given that endogenous neurotrophic factors such as BDNF and cilliary neurotrophic factor (CNTF) have been shown to be essential for enhancing regeneration [12-15].

Recently, we and others have demonstrated that after SNI, there is a robust macrophage response infiltrating DRGs [16] and even other parts of CNS such as the spinal cord [17] and optic nerve [18]. In addition, further evidence from our laboratory indicates that macrophages can express a number of neurotrophic factors such as BDNF, NGF, and other neurotrophic cytokines [19]. In this study, we hypothesized that macrophage cells play a crucial role in the conditioning and regeneration of the CNS-DRG branch (i.e. afferent fibres). To test this, we selectively and temporarily depleted macrophage cells during the conditioning phase of DRG neurons (i.e. prior to, during and after SNI) via the intravenous delivery of a liposome-encapsulated clodronate, a technique described to result in macrophage suicide [20,21]. We found that the temporal depletion of these cells completely abolished the regenerative trigger characteristic of this model and therefore, we propose a beneficial role of macrophage cells in the regeneration of pre-conditioned DRG neurons.

## Methods

### Animals

Adult female Sprague Dawley (SD) rats (10-12 weeks) were used under the guidelines of the National Health and Medical Research Council of Australia and approved by the Animal Welfare Committee of Flinders University of South Australia.

### Treatment Groups

Animals were divided into 2 experimental groups, both of which received a SNI (day 0) followed by a dorsal column cut (DCC, day 7). The timing of the CNS injury relates to the optimal conditioning of DRG neurons, known to occur seven days after SNI, resulting in the maximum amount of CNS regeneration possible [7]. Control animals (n = 10) received intravenous (iv) tail vein injections of sterile saline alone (2 ml/injection). Test animals (n = 10) received iv tail vein injections of liposome-encapsulated clodronate (2 ml/injection). In both of these two groups sterile saline was administered as the vehicle during iv delivery and the only difference between them was the use of liposomes in the treatment group. Note that control animals did not receive liposome-encapsulating saline given the possibility that liposomes alone may "block macrophage phagocytosis by saturation and may suppress or activate other macrophage functions" [22]. Consequently, this would have provided inconsistent comparisons between liposome-treated and control group animals.

### Sciatic Nerve Injury (SNI)

For all surgical procedures, the toepinch-reflex test was used to determine effectiveness of the anaesthetic prior to surgery using a mixture of ketamine (100 mg/kg) and xylazine (100 mg/kg) delivered intraperitoneally. Briefly, a primary longitudinal cut was made on the skin overlaying the femur of the left hind limb. The incision was extended proximally and distally exposing the thigh muscle. Sharp surgical scissors were inserted and opened into the muscle through the first layer to the level at which the sciatic nerve runs. After locating the branching of the sciatic nerve, the nerve was ligated proximal to its trifurcation and cut below the ligation site with fine surgical scissors. The wound was sutured closed using a 6/0 surgical silk suture and animals were placed into individual cages.

### Dorsal Column Cut (DCC)

After laminectomy at T9-T10 and a small incision in the dura mater, the dorsal columns of the spinal cord were crushed with iris scissors inserted at a depth of approximately 1.5-2 mm (marked on the scissors' tip) [23]. A sharp scalpel blade was passed through the wound twice to confirm bilateral DCC [24]. A small piece of gelfoam was temporarily placed over the lesion site to encourage blood clotting and the overlaying muscles were sewn together with a 6/0 surgical suture. The skin was stapled closed and the animals were returned to their cages. After surgery, animals were housed separately and received subcutaneous injections of the analgesic drug buprenorphine (0.03 mg/kg) for a period of up to 5 days to alleviate postoperative pain. For all spinal cord injured animals manual bladder expression was performed two to three times per day and antibiotics were administered if required [25].

### Retrograde Tracer Injection

To investigate the presence of ascending regenerated fibres in the injured spinal cord (i.e. across the injury epicentre), a somatic retrograde tracer Fast Blue (FB, 5% in saline, Sigma) was injected into the dorsal column of the proximal stump, 3-4 mm rostral from the site of spinal cord injury (SCI; injection depth 1-1.1 mm, total volume delivered 0.1 μl). FB was administered 2 weeks after CNS lesion, using a stereotaxic frame and a pulled glass micropipette needle. Note that to alleviate any pain caused by tracer injections into the spinal cord, all animals received injections of buprenorphine (0.03 mg/kg) for up to 3 days after injection.

### Anterograde Tracer Injection

To further examine the presence of ascending CNS fibres regenerating across the SCI epicentre, the anterograde tracer Biotinylated Dextran Amine (BDA, 10% in saline, 10000 mw, Molecular Probes) was injected into the dorsal column in the lumbar region of the spinal cord [26-28]. BDA was delivered 2 weeks after CNS lesion as described for retrograde tracing (injection depth 1-1.1 mm, total volume delivered 0.1 μl). All care was taken to keep tracer injections within the dorsal column of the spinal cord. Some injected cords were randomly selected to confirm tracer deposition, however, not all injections sites were examined and further damage to the cord at these sites was not investigated in this study.

### Liposome Preparation and Administration

Liposomes were prepared according to previous studies [29-32]. Briefly, 86 mg of egg phosphatidylcholine and 8 mg of cholesterol were dissolved in 5 ml of chloroform in a round-bottom flask. Chloroform was removed by using a low-vacuum rotary evaporator at 37°C to form a thin lipid film around the flask. The lipid was then dispersed with 10 ml sterile phosphate buffered saline (PBS, 0.1 M, pH 7.4) containing 2.5 gm of clodronate (dichloromethylene-diphosphonate-DMDP, Sigma) and incubated on a gentle stirrer at room temperature (RT, 2 hrs). After incubation, the suspension was sonicated (50 Hz) at RT (3 min) and incubated again at RT with no stirring to allow for liposome formation (2 hrs). Liposomes were centrifuged at 10,000 g (15 min) at RT to remove any free clodronate. The remaining pellet was washed twice in sterile PBS at 20,000 g at RT (30 min) and resuspended in 4 ml sterile PBS to be used immediately [29,30,33].

### Liposome Administration

For all test animals, liposomal delivery was administered on three separate occasions, once: i) three days prior to; ii) immediately after; and iii) four days after SNI. The specific timing of liposome administration ensured macrophage depletion commencing 3 days prior to SNI and ending 3 days after DCC. It is known that macrophage cells are depleted within 24 hrs after liposomal treatment and begin to slowly repopulate approximately 5-7 days after last administration of clodronate (personal communication with liposome pioneer Dr Nico van Rooijen, Netherlands). No adverse side effects were observed in any of the liposome-treated animals.

### Perfusion and Cryosectioning

All animals were injected intraperitoneally with 5% chloral hydrate in distilled water and perfused transcardially with 1% NaNO_2_/phosphate buffer (PB, 0.1 M, pH 7.4) followed by a 4% paraformaldehyde (PFA)/PB. Perfusions were performed 4 weeks after the CNS lesion with all dissected tissues post-fixed in 4% PFA and cryoprotected in 30% sucrose/PB at 4°C (48 hrs). Spleens were cryosectioned at 20 μm (cross-sections), DRG at 20 μm (coronal sections) and spinal cords at 40 μm (longitudinal sections). All specimens were mounted on 2% gelatine-coated glass slides.

### Immunohistochemistry (IHC)

For IHC, DRG and spinal cord sections were washed in 0.5% H_2_O_2_/50% ethanol at RT (30 min) to quench endogenous peroxide activity, rinsed in PBS, followed by three washes in PBS containing 1% Tween-20 detergent (PBST). These sections were blocked in 20% normal horse serum (NHS, 2 hrs) before incubation with primary antibodies at 4°C (48 hrs). The primary antibodies used were: mouse-anti-rat cluster differentiation 68 (CD68, macrophage, 1:400, Serotec), rabbit-anti-glial fibrillary acidic protein (GFAP, astrocyte/satellite cells, 1:500, Dako). A combination of the following secondary antibodies for single and/or double labelling included: sheep-anti-mouse-cy3-IgG (1:500, Jackson), donkey-anti-mouse-cy2-IgG (1:500, Jackson), donkey-anti-rabbit-488-IgG (1:500, Jackson), sheep-anti-rabbit-cy3-IgG (1:500, Jackson). The specificity of the observed IHC procedure was validated by omitting the primary antibody and/or by using a non-immune serum instead of the primary antibody [24].

### Immunoperoxidase Staining

BDA injected tissue was treated in 3% H_2_O_2_/100% methanol at RT (10 min), rehydrated in PBS and thoroughly washed in PBST. This was followed by incubation with streptavidin-HRP conjugated antibody (1:2000, Vector Laboratories) in PBST at RT (60 min). After extensive washing with PBST, sections were developed in a solution containing 0.05% 3'3-diaminobenzidine tetrahydrochloride (DAB, Sigma), 0.06% NiSO_4 _and 0.005% glucose oxidase [26,34].

### Relative DRG Somatic Count

The relative number of retrograde labelled FB^+ ^DRG neurons was obtained by serially counting every fourth section from the ipsilateral and contralateral L4 DRG (total of 5 sections/animal, n = 10). This method of counting allowed for the relative estimation of FB labelled cell bodies and to avoid the possibility of double counting, only those neurons with visible nuclei were counted [35,36]. Note that in all instances, FB^+ ^neurons were considered regenerated neurons [24].

### Quantification of Ascending Fibre Regeneration

The number of BDA-labelled fibres was counted at both stumps of the spinal cord including the lesion epicentre (0 mm) and 1 mm rostral and caudal. As we have previously described [37], the axon index was calculated as a percentage of every fourth section (total of 10 sections/animal, n = 6). Note that no BDA-labelled fibres were found rostrally and/or at the epicentre in liposome-treated animals.

### Cellular Quantification Method

Due to the complexity in identifying individual populations of macrophage cells (CD68^+^) and astrocytes (GFAP^+^) present in the spinal cord after injury, we determined the percentage area fraction of the section occupied by these stained structures [31,38,39]. Briefly, using ImageJ (image processing program, NIH version 1.37) 20× (697.68 × 522.72 μm) magnification images immunostained against the aforementioned antibodies were converted to binary contrast images (black and white). This provided a threshold by subtracting background levels from the immunoreactive stained areas and allowed the determination of the percentage area fraction per image to be collected, tabulated and statistically analysed [22,38,40,41].

### Sandwich BDNF ELISA Test

According to manufacturer's instructions (Millipore.com), serum samples collected at the end of the experimental period were analysed for BDNF concentration level using an ELISA kit. Briefly, samples were incubated overnight at 2-4°C in BDNF ELISA plates pre-coated with rabbit anti-human BDNF polyclonal antibody. After incubation, plates were thoroughly washed and incubated at RT (2-3 hrs) with a biotinylated mouse anti-human BDNF monoclonal antibody (1:1000). Plates were then washed and incubated at RT (60 min) with a streptavidin-HRP conjugate (1:1000), washed again, developed with a TMB/E substrate at RT (15 min). The optical density (OD) was measured at 450 nm and plotted on a standard curve.

### Statistics

In all graphs, columns represent an averaged mean (n = 10 and/or as specified per figure) and error bars indicate standard error of mean (+/- S.E.). Comparisons between groups were made using an independent samples t-test. Results were considered significant if P < 0.05.

## Results

### Effectiveness of Macrophage Depletion

**Figure 1:**
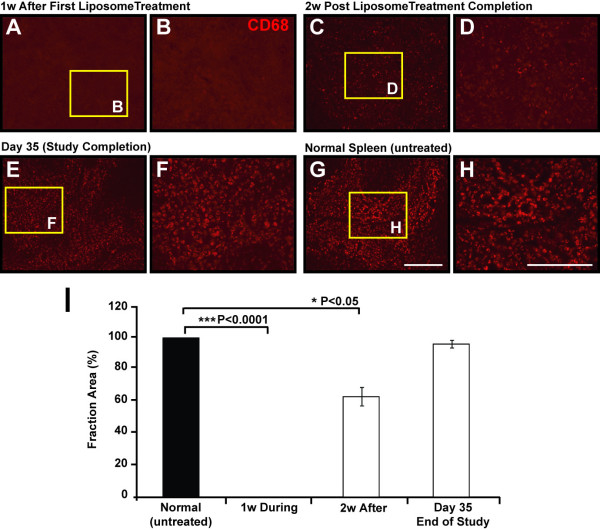
**Confirmation of Macrophage Cell Depletion Examined in the Spleens of Liposome-Treated Animals**. **A-B) **1 week after liposome administration no macrophage cells were present in the spleens of treated animals, as confirmed by the absence of CD68 (macrophage marker) immunoreactivity (***P < 0.0001; **I**). **C-D) **By 2 week after liposome treatment completion, macrophage cells (CD68^+^) had already began to repopulate in the spleen of treated animals (*P < 0.05; **I**). **E-F) **At the end of the experimental period (day 35) macrophage numbers in the spleen of treated animals had returned to normal levels, as compared to untreated animals (**G-H**). **I**) Quantification of immunoreactive CD68^+ ^cells as shown in panels **A-H**, demonstrated a significant cellular reduction during liposome administration and during repopulation 2 weeks after treatment completion. No differences in macrophage cells were found at the end of the study. Columns represent an averaged mean (5 sections per animal, n = 5) and error bars indicate error of mean (+/- S.E.). Scale bars A, C, E, G 500 μm, enlarged views B, D, F, H 200 μm.

IHC analysis of spleen sections immunostained to detect macrophage cells (CD68^+^) were used to determine the efficacy of macrophage depletion using clodronate liposomes [22]. Our results revealed a significant difference in macrophage numbers during liposome treatment (1 week after first injection), indicative of successful cellular depletion (P < 0.0001; Figure 1A-B &1I). Spleens tested 2 weeks after liposome treatment completion showed early signs of macrophage repopulation evident by CD68^+ ^immunoreactivity in spleens (Figure 1C-D &1I). However, macrophage numbers had not reached normal levels (P < 0.05; Fig. 1I). Comparatively, at the end of the experimental period (day 35) we found a complete repopulation of macrophage cells in spleens of liposome-treated animals, as compared to untreated naïve spleens (Figure 1E-H &1I).

### Retrograde Tracing in DRG

**Figure 2:**
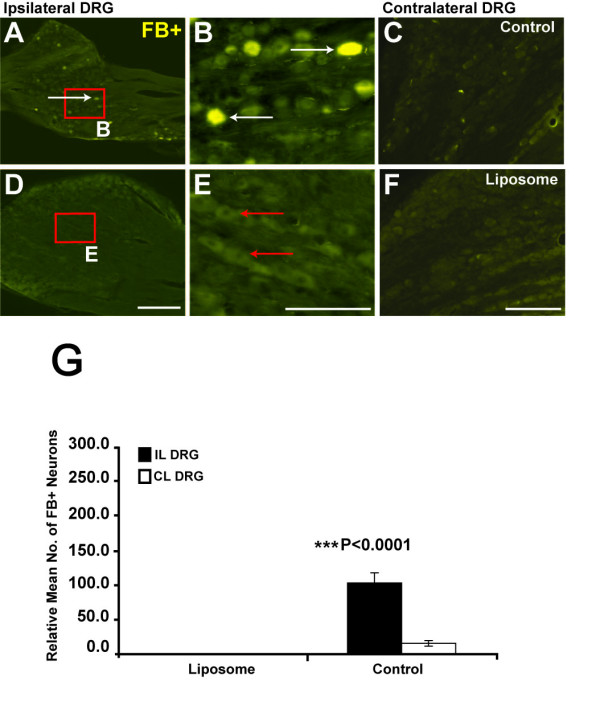
**No Regenerated FB^+ ^DRG Neurons Found in Liposome-Treated Animals**. **A-B) **In the ipsilateral DRG, only saline-treated control animals showed retrograde labelled FB^+ ^neuronal cell bodies (white arrows), as compared to the contralateral DRG (**C**). **D-E**) No FB labelled neurons were found in any of the liposome-treated animals, in the ipsilateral (red arrows) or contralateral DRG (**F**). **G) **Quantification of FB^+ ^cell bodies in ipsilateral (black bar) and contralateral (white bar) DRGs revealed significantly more labelled neurons in saline-treated animals, as compared to unlabelled DRG neurons in liposome-treated (***P < 0.0001). Note that the contralateral DRG was used from the uninjured side as a control. Columns represent an averaged mean (5 sections per animal, n = 10) and error bars indicate error of mean (+/- S.E.). FB = fast blue tracer, DRG = dorsal root ganglia, CL = contralateral, IL = ipsilateral. Scale bars A and D 500 μm, C and F 300 enlarged views 200 μm.

Quantification of retrograde labelled FB^+ ^neurons in the ipsilateral L4 DRG revealed some interesting variations between test and control groups. Consistent with previous observations [7], our results indicated positive retrograde labelling only in the ipsilateral DRG of saline-treated control animals (white arrows, Figure 2A-B), as compared to neurons in the contralateral DRGs (Figure 2C). However, no FB^+ ^labelled cell bodies were found in the ipsilateral and contralateral DRGs of liposome-treated animals (red arrows, Figure 2D-F). Statistically, the presence of FB^+ ^labelled neurons in the control group as compared to unlabelled DRG neurons in the liposome-treated group indicated a lack of retrograde labelling in the latter group (P < 0.001; Figure 2G). These results are indicative of poor attempts at axonal regeneration, possibly attributable to the effects of liposome administration and temporal depletion of macrophage cells.

It seems unlikely that tracer leakage could have affected retrograde labelling of DRG neurons in all of the liposome-treated animals given that this would have resulted in labelled cell bodies in both ipsilateral and contralateral DRG, which was not found (Figure 2G). Note that in all instances, the contralateral DRG was used as the control (uninjured) side. The possibility that regenerated fibres could still be present in the spinal cord of liposome-treated animals was further explored using anterograde tracing.

### Anterograde Tracing in Spinal Cord

**Figure 3:**
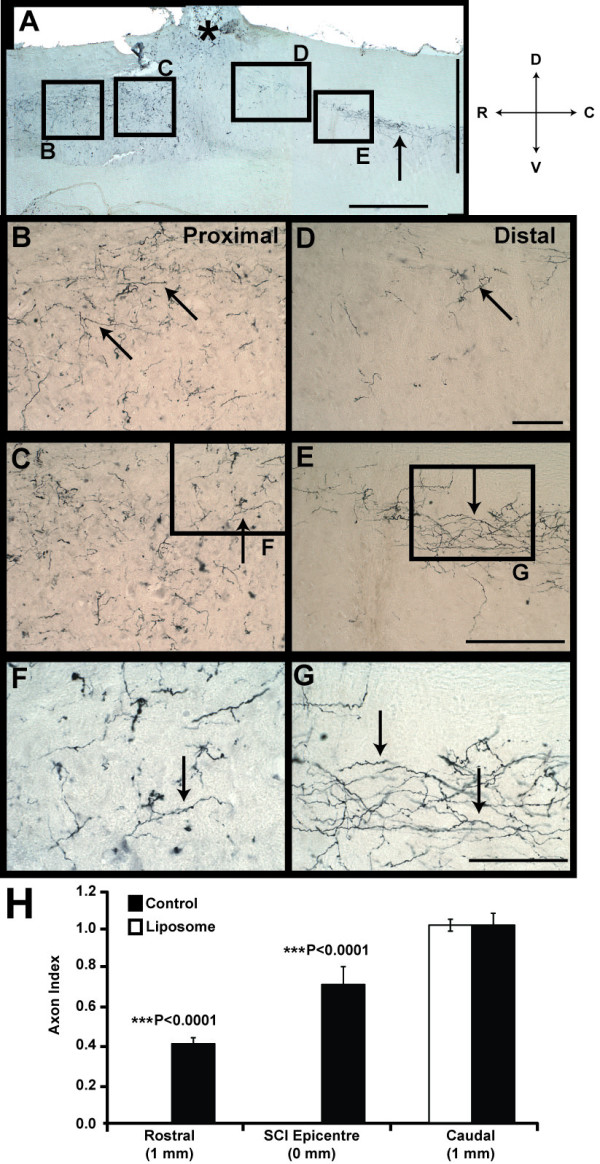
**Anterograde BDA-Labelled Fibres in the Spinal Cord of Saline-Treated Animals**. **A) **Montage of SCI epicentre (black asterisk) demonstrating extensive anterograde labelling of ascending fibres in both proximal and distal stumps of the injured spinal cord (black arrows). **B-G) **Higher magnification images confirming the presence of BDA-labelled fibres extending across both stumps (black arrows), consistent with previous retrograde labelled results described in Figure 2. **H) **Quantification of percentage of BDA-labelled fibres revealed the presence of regenerated ascending fibres found 1 mm rostral and at the injury epicentre (0 mm) in control animals only (***P < 0.0001). Similar number of labelled fibres was found 1 mm caudal in the spinal cord of control and liposome-treated animals. BDA = biotinylated dextran amine, DCC = dorsal column cut. Directional key: D = dorsal, V = ventral, C = caudal, R = rostral. Scale bars montage 800 μm (horizontal bar) and 1 mm (vertical bar), B -E 200 μm, F-G enlarged views 100 μm.

**Figure 4:**
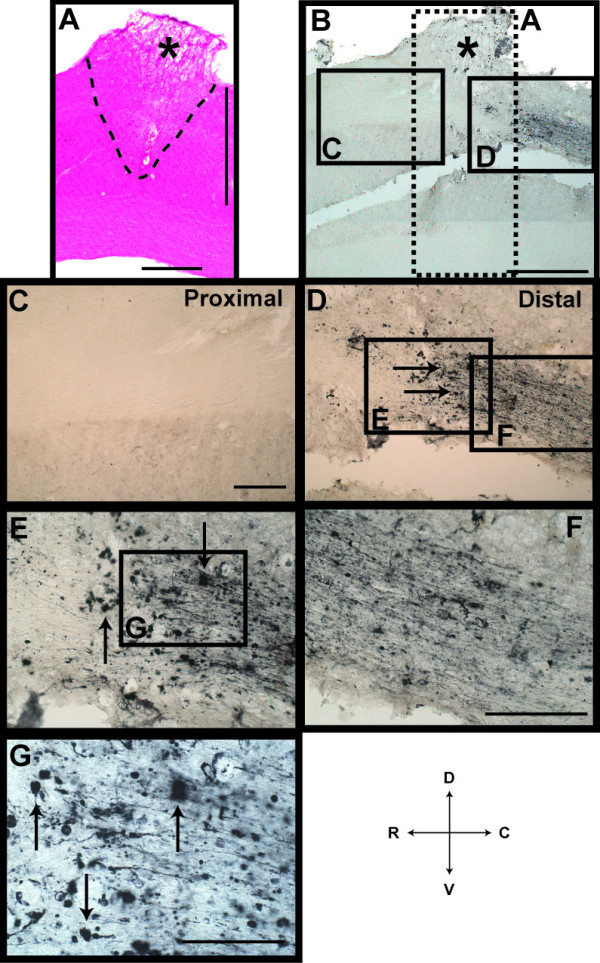
**Anterograde BDA-Labelled Fibres in the Spinal Cord of Liposome-Treated Animals**. **A) **Histology-processed section through the SCI epicentre (black asterisk) revealed extent of lesioning through the dorsal columns (dashed line; shown as insert in panel B). **B) **Montage of lesion site (black asterisk) demonstrating anterograde BDA-labelled fibres present only in the distal stump of the spinal cord (**D**), with no visible fibres found in the proximal stump (**C**). **E-G**) Closer observation distally revealed extensive neuronal collapse and retraction of BDA-labelled fibres evident by their bulb-like structure (black arrows). BDA = biotinylated dextran amine, DCC = dorsal column cut. Directional key: D = dorsal, V = ventral, C = caudal, R = rostral. Scale bars, A (horizontal bar) 500 μm and (vertical bar) 1 mm, montage B 800 μm, C-E 200 μm, F-G enlarged views 100 μm.

Normally in the pre-conditioned lesion model, regenerated ascending afferent fibres extend through the dorsal column of the spinal cord [7]. Here, we assessed this by anterograde BDA labelling of fibres in the dorsal column of the injured spinal cord. Results from saline-treated control animals demonstrated the presence of ascending afferent fibres extending rostrally from the distal stump, across the DCC and into the rostral stump (Figure 3A-H). Quantification of BDA-labelled fibres 1 mm caudal revealed similar number of ascending fibres close to site of injury in both experimental groups, however, no regenerated fibres were found in liposome-treated animals at the lesion epicentre and 1 mm rostral (P < 0.0001; Figure 3H**&**Figure 4). Evidently, in the liposome-treated group labelled fibres in the dorsal column halted at the site of lesion and did not cross the SCI epicentre (Figure 4A-G). Higher magnification images of BDA-labelled fibres in the caudal stump, revealed fibre retraction and collapse close to the site of injury, with no ascending fibres found in the rostral stump (Figure 4C). The lack of regenerated fibres across the SCI epicentre was consistent with the absence of retrograde labelled FB^+ ^DRG neurons within this group. This finding confirmed previous assumptions relating to abortive attempts at axonal regeneration and offers tangible explanations for the role of macrophage cells in the pre-conditioned model of injury.

### Macrophage and Astrocyte Quantification

**Figure 5:**
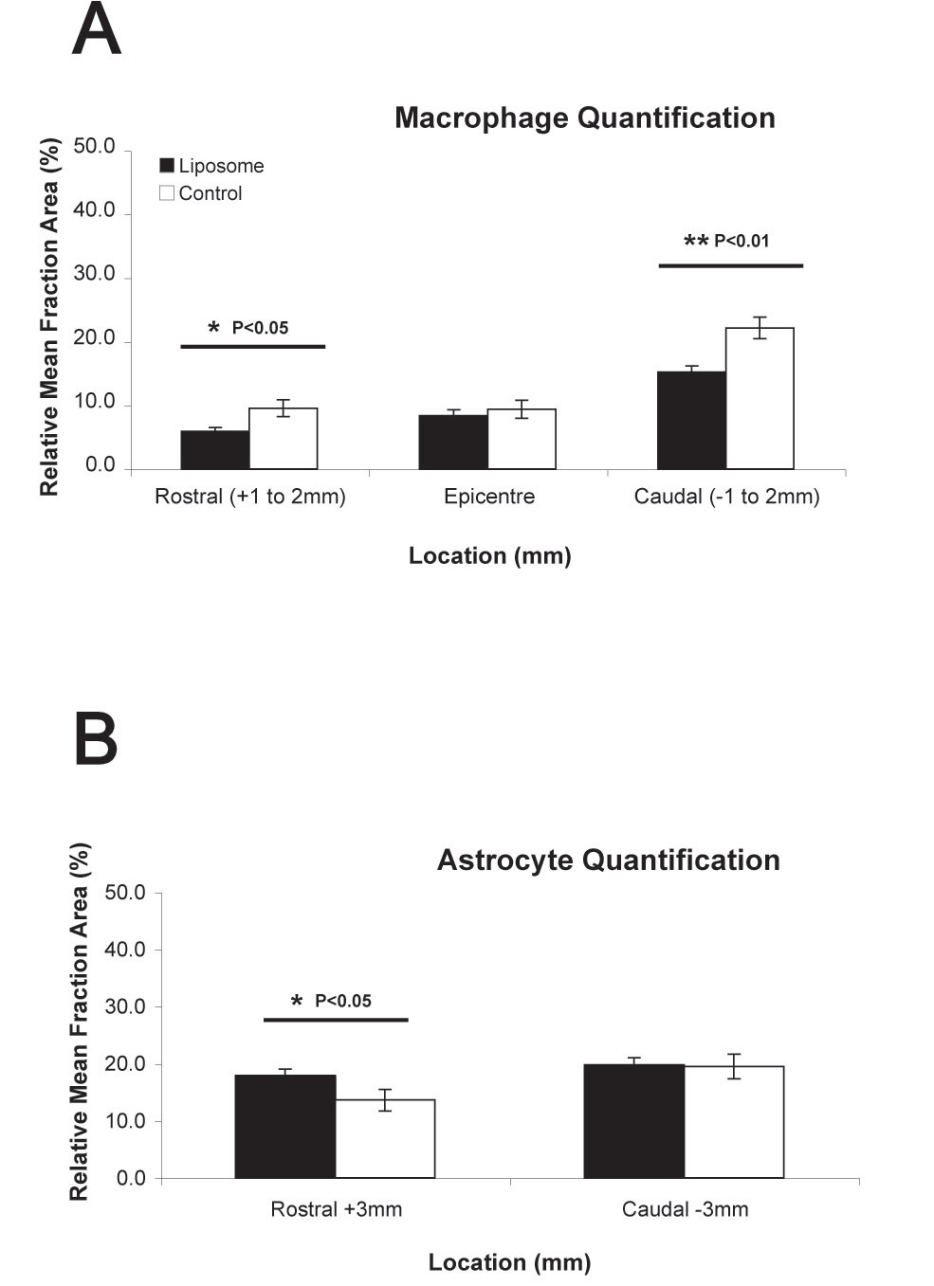
**Macrophage and Astrocyte Quantification in the Injured Spinal Cord**. **A) **Statistical analysis of macrophage numbers in the spinal cord of both treatment groups indicated a greater macrophage presence in control animals than in liposome-treated. Specifically, in control animals these numbers were significantly higher 1-2 mm both rostral and caudal from the SCI epicentre, as compared to liposome-treated animals (*P < 0.05, **P < 0.01, respectively), while no differences were found at the SCI epicentre. **B) **Astrocyte quantification 3 mm rostral from the lesion epicentre revealed a greater reduction in astrocyte activation in control animals (*P < 0.05). No differences were observed between groups caudally. Columns represent an averaged mean (n = 10) and error bars indicate error of mean (+/- S.E.).

Macrophage quantification at 1-2 mm both rostral and caudal from the SCI epicentre revealed greater macrophage numbers (CD68^+^) in the saline-treated group, as compared to the liposome-treated group 4 weeks after CNS lesion (P < 0.05 & P < 0.01, respectively; Figure 5A). No statistically significant difference in macrophage numbers between these two groups was found at the SCI epicentre. Similarly, astrocyte quantification (GFAP^+^) 3 mm caudal from the lesion site reached similar levels with no statistical differences found between both groups (Figure 5B). Conversely, 3 mm rostral from the lesion site we found a significantly higher astrocyte expression (GFAP^+^) in liposome-treated animals as compared to controls (P < 0.05).

### BDNF ELISA Serum Levels

**Figure 6:**
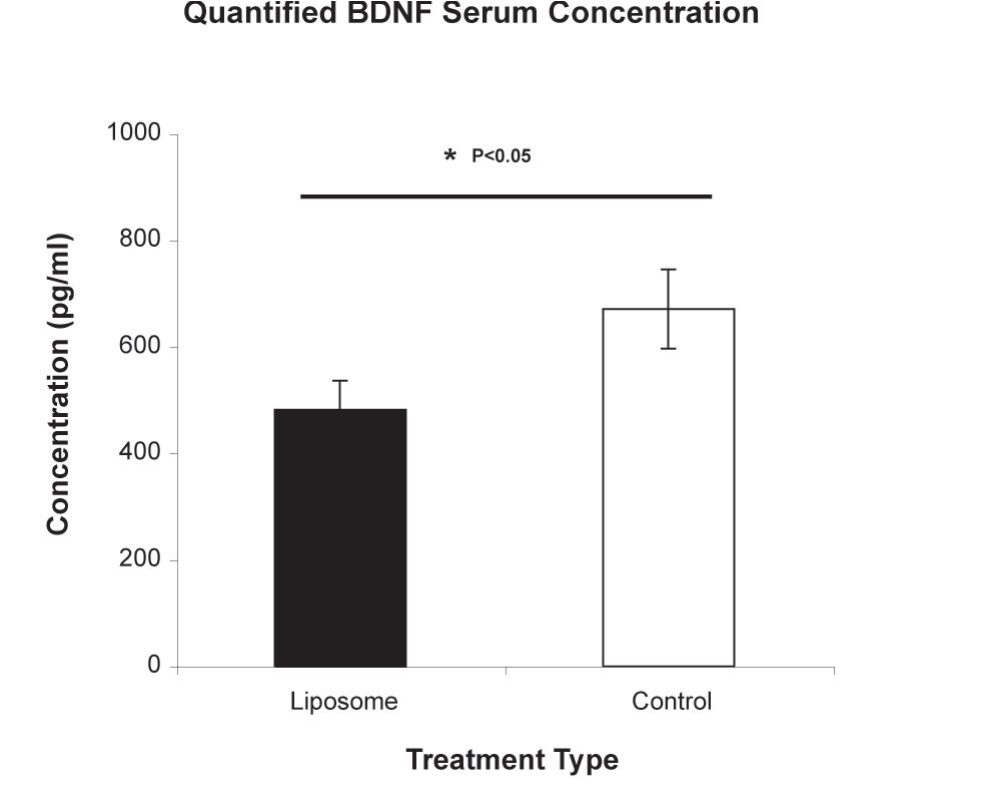
**Higher BDNF Concentration Serum Levels in Saline-Treated Controls**. Analysis of serum trophic levels at the end of the experimental period revealed an increased BDNF serum concentration in saline-treated controls, as compared to liposome-treated animals (*P < 0.05). Columns represent an averaged mean (n = 10) and error bars indicate error of mean (+/- S.E.). BDNF = brain derived neurotrophic factor.

Calculated serum levels from both treatment groups revealed a significantly higher BDNF concentration level in control animals than in liposome-treated (P < 0.05; Figure 6). The fact that immune cells, such as macrophage cells have been reported to support neurite outgrowth by secreting neurotrophic factors such as BDNF [15,42,43], provide a plausible explanation for the lack of regenerated fibres in liposome-treated animals.

## Discussion

### Effects of Macrophage Depletion

The conditioning SNI normally supports the regeneration of CNS afferent fibres of adult DRG neurons [7]. However, we found that the temporal depletion of macrophage cells during the conditioning phase of DRG neurons, consequently abolished the regenerative competence of injured CNS fibres.

Comparisons between injury-matched control and liposome-treated animals revealed a considerable lack of regeneration in the latter group, when compared to retrogradely labelled FB^+ ^DRG neurons found in control animals. Concomitantly, anterograde tracing of ascending CNS fibres demonstrated extensive axonal collapse and retraction by the formation of bulb-like structures at the end of axons in liposome-treated animals. In contrast, lengthy BDA-labelled afferent fibres were found in the proximal stump of saline-treated control animals. In addition, astrocyte quantification in liposome-treated animals revealed a higher astrocytic expression level rostral to the spinal cord lesion. This level of astrocyte expression most likely represents the amount of glial scar formation (refer to additional file 1), which may be associated with the observed axonal collapse, as it forms an inhibitory barrier against axonal regrowth [44-46].

Furthermore, macrophage quantification revealed greater numbers in control animals, specifically in regions rostral and caudal to the lesion, as compared to liposome-treated animals. Note that, we do not attribute this variation in macrophage numbers solely to the depletion of these cells, given that: (i) spleens of liposome-treated animals showed macrophage repopulation returned to normal levels; and (ii) liposomal treatment was only temporarily administered, allowing sufficient time for macrophage cells to infiltrate the CNS lesion. This suggests that macrophage presence during the conditioning phase following SNI, is critical for the early activation and guidance of these cells into the CNS compartment [17].

**Figure 7:**
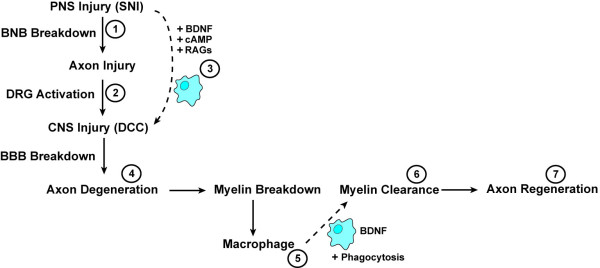
**Proposed Mechanistic Model for the Regeneration of Ascending Fibres in Pre-Conditioned DRG Neurons**. Regenerative competence of pre-conditioned DRG neurons is described as a multi-faceted cascade of events: (**1-2**) SNI results in DRG and macrophage activation, upregulation of BDNF, cAMP and regeneration-associated genes (RAGs); all contributing to the beneficial phenotypic 'priming' of macrophage cells. (**3**) Facilitated entry of primed macrophage cells into the CNS compartment prior to CNS lesion. (**4**) SCI resulting in axonal injury, axon-myelin breakdown associated with the process of Wallerian degeneration. (**5-6**) Efficient removal of axonal and myelin debris by BDNF expressing macrophages beneficially activated with enhanced phagocytic capabilities, (**7**) consequently leading to axonal regeneration of the injured CNS-DRG branch. BNB = blood nerve barrier, RAGs = regeneration-associated genes, BBB = brain-blood barrier.

Moreover, BDNF analysis of blood samples demonstrated elevated serum concentration levels in control animals. This difference in endogenous BDNF availability, at least for injured peripheral nerves has been demonstrated to support axonal regeneration and myelination following trauma [47], and is likely to be also contributing to the regeneration of ascending afferent fibres [24]. Certainly, injury to the peripheral DRG branch may be stimulating macrophage cells with a phenotype beneficial for CNS regeneration, as demonstrated using peripheral nerve fragments, reported to enhance phagocytosis and secretion of BDNF [48,49]. BDNF is also a nerve trophic factor recently confirmed by our laboratory to be synthesized by macrophages [19] and is a potential mechanistic factor in the successful regeneration of matured CNS neurons after a pre-conditioned lesion (Figure 7).

### Macrophage Contribution

The recruitment number and the phagocytic activity of macrophages has been previously shown to improve the clearance of myelin debris [50], as well as provide important signaling molecules for the improved regeneration of injured axons, dependant on the timing of macrophage activation [51]. This is important given that myelin clearance during Wallerian degeneration by macrophage cells has been reported to be one of the major differences between the PNS and the CNS [52,53]. It is also noteworthy that SNI induces circulating macrophage cell infiltration into the uninjured spinal cord, where these cells proliferated and differentiated into microglia cells [17]. This highlights another interesting factor in relation to immune surveillance into the uninjured CNS compartment as it introduces new functions in "bi-directional communication between the CNS and immune system" [54]. Mechanistically, we believe the regenerative trigger characteristic of this model illustrates a complex neuroimmune interaction between the peripheral and central nervous system, regardless of the immune privileged status of the CNS [55,56].

## Conclusions

Despite of the controversy of macrophage cells in CNS repair [57,58], here we ascribe a beneficial role for inflammatory cells in CNS regeneration, given that *in vivo *macrophage depletion led to astrocyte up-regulation, reduced macrophage infiltration into the CNS and a down-regulation in endogenous BDNF serum concentration. Our data suggest that macrophage activation might be playing a role in the conditioning effect on the regeneration of DRG neurons. These cells are highly attractive for promoting CNS repair, as long as the pro-regenerative process is not coupled to undesired inflammation.

## Competing interests

The authors declare that they have no competing interests.

## Authors' contributions

EAS carried out all surgical procedure, liposome preparation, IHC, writing of manuscript. ANP carried out statistical analysis, figure preparation and some IHC. MS constructed study design and critical corrections of manuscript. XFZ carried out study design, guidance and drafting of manuscript. All authors read and approved the final manuscript.

## Supplementary Material

Additional file 1 **Glial Scar Formation at Spinal Cord Injury Epicentre**. Montage of the spinal cord lesion epicentre demonstrating the extent of glial scar formation after the injury to the dorsal columns. Immunoreactive GFAP^+ ^staining identifies the location of lesion epicentre (yellow asterisk), validates the paradigm for astrocyte quantification and delineates the presence of the physical/biochemical barrier against axonal regeneration in the matured CNS (dashed line). Scale bars montage 500 μm. Click here for file
