# Supplementary figures and images for: Macrophage presence is essential for the regeneration of ascending afferent fibres following a conditioning sciatic nerve lesion in adult rats
Source: BMC Neurosci. 2011 Jan 20;12:11. doi: 10.1186/1471-2202-12-11 (PMC3039622; doi:10.1186/1471-2202-12-11)

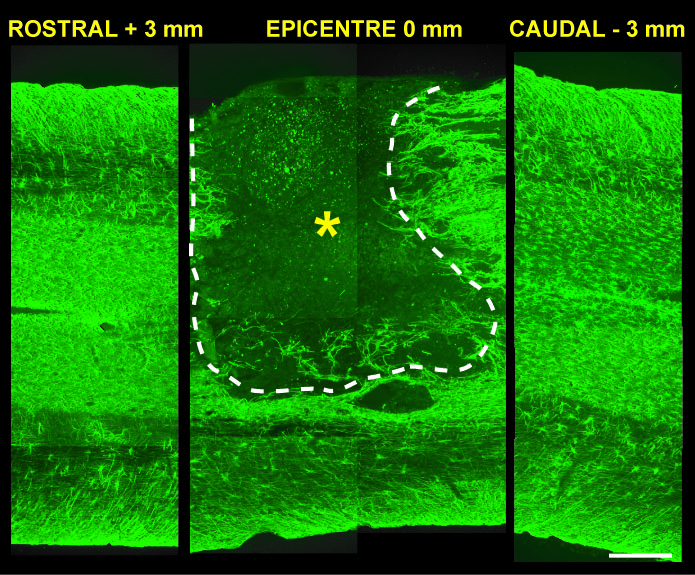

Supplement: Additional file 1 — Glial Scar Formation at Spinal Cord Injury Epicentre. Montage of the spinal cord lesion epicentre demonstrating the extent of glial scar formation after the injury to the dorsal columns. Immunoreactive GFAP+ staining identifies the location of lesion epicentre (yellow asterisk), validates the paradigm for astrocyte quantification and delineates the presence of the physical/biochemical barrier against axonal regeneration in the matured CNS (dashed line). Scale bars montage 500 μm. [file 1471-2202-12-11-S1.JPEG]
